# Semantic and traditional feature fusion for software defect prediction using hybrid deep learning model

**DOI:** 10.1038/s41598-024-65639-4

**Published:** 2024-07-01

**Authors:** Ahmed Abdu, Zhengjun Zhai, Hakim A. Abdo, Redhwan Algabri, Mohammed A. Al-masni, Mannan Saeed Muhammad, Yeong Hyeon Gu

**Affiliations:** 1 School of Software, Northwestern Polytechnical University, Xi’an, 710072 China; 2 School of Computer Science, Northwestern Polytechnical University, Xi’an, 710072 China; 3 School of Computer Science, Dr.Babasaheb Ambedkar Marathwada University, Aurangabad, India; 4 Research Institute of Engineering and Technology, Hanyang University, Ansan, 15588 Korea; 5 Department of Artificial Intelligence and Data Science, College of Software and Convergence Technology, Sejong University, Seoul, 05006 Republic of Korea; 6 Department of AI and Robotics, Sejong University, Seoul, 05006 South Korea

**Keywords:** Software, Computer science

## Abstract

Software defect prediction aims to find a reliable method for predicting defects in a particular software project and assisting software engineers in allocating limited resources to release high-quality software products. While most earlier research has concentrated on employing traditional features, current methodologies are increasingly directed toward extracting semantic features from source code. Traditional features often fall short in identifying semantic differences within programs, differences that are essential for the development of reliable and effective prediction models. In contrast, semantic features cannot present statistical metrics about the source code, such as the code size and complexity. Thus, using only one kind of feature negatively affects prediction performance. To bridge the gap between the traditional and semantic features, we propose a novel defect prediction model that integrates traditional and semantic features using a hybrid deep learning approach to address this limitation. Specifically, our model employs a hybrid CNN-MLP classifier: the convolutional neural network (CNN) processes semantic features extracted from projects’ abstract syntax trees (ASTs) using Word2vec. In contrast, the traditional features extracted from the dataset repository are processed by a multilayer perceptron (MLP). Outputs of CNN and MLP are then integrated and fed into a fully connected layer for defect prediction. Extensive experiments are conducted on various open-source projects to validate CNN-MLP’s effectiveness. Experimental results indicate that CNN-MLP can significantly enhance defect prediction performance. Furthermore, CNN-MLP’s improvements outperform existing methods in non-effort-aware and effort-aware cases.

## Introduction

Software defect prediction (SDP) approaches become more critical as the scale and complexity of software projects grow. SDP helps developers and testers identify potential defects in a software project early in its lifecycle, effectively managing test resources, enhancing the testing process, and improving software quality^1–3^.

Earlier on, researchers used methods based on traditional features to solve the problem, relying on different data types, such as previous defects, code metrics (lines of code, complexity), or process metrics (recent activity, number of changes). Subramanyam and Krishnan ^4^ presented empirical evidence for using object-oriented (OO) design complexity measures in determining software defects, such as the Chidamber and Kemerer (CK) set. Moser et al.^5^ assessed the effectiveness of static code features and change metrics in predicting software defects. Hassan^6^ created an SDP model using complexity measures based on the change of source code rather than the source code itself. In some cases, the researchers’ conclusions were inconsistent; for example, in opposition to the decision of Fenton and Ohlsson ^7^, Gyim’othy et al.^8^ showed positive outcomes in the case of using size metrics. Yang et al.^9^ suggested a SDP model for Just-in-Time (JIT) defect prediction. Their approach uses a deep belief network (DBN) to extract an informative set of features from a given set of basic change metrics. Subsequently, it utilizes logistic regression to train a classifier model based on the extracted features.

As an alternative to methodologies based on traditional metrics, strategies based on semantic features have evolved for defect prediction in recent years. This method predicts defects directly from the project’s source code rather than metric generator analysis. To leverage the semantic information of the source code, Wang et al.^10^ utilized a DBN to derive semantic insights from token sequences obtained from the ASTs of the source code. Meanwhile, Phan et al.^11^ created a control flow graph (CFG) from a program’s assembly code and employed CNN to analyze the program’s semantic characteristics. Diverging from these approaches, Dam et al.^12^ proposed a tree-based strategy for source code defect prediction, transforming various levels of AST nodes into vectors with actual values. These vectors were then processed using long short-term memory (LSTM) for identifying software defects. Additionally, Majd et al.^13^ developed an SDP model that operates at the statement level, utilizing LSTM. Their research contributed metrics that evaluate the complexity of code statements at this granularity.

**Figure 1:**
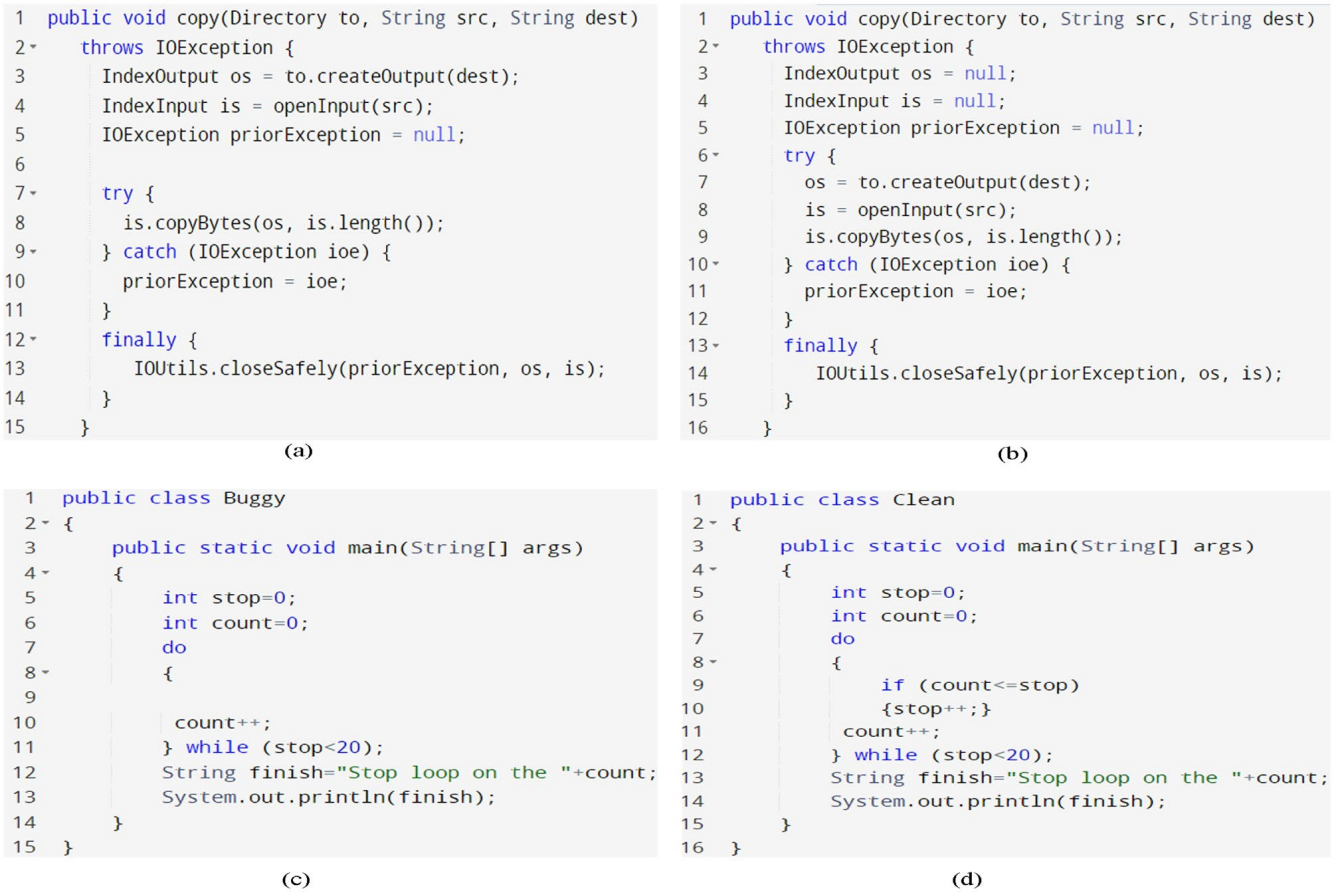
Motivating example: (**a**) Original defective code with a memory leak, (**b**) Code after fixing the defective, (**c**) Original defective code with an infinite loop, and (**d**) Code after fixing the defective.

Previous research in Source Code Defect Prediction (SDP) has predominantly utilized two types of features: semantic and traditional. Semantic features can capture the contextual nuances of source code, nuances beyond the expressive capacity of traditional features. For example, consider Fig. 1 which illustrates four Java code files; Fig. 1-a presents an original version with a defect (a memory leak bug), whereas Fig. 1b depicts a rectified version of the code (after the bug has been addressed). In Fig. 1a, the code exhibits an IOException due to the initialization of variables (’is’ and ’os’) before the try block. This coding error could result in a memory leak, a problem rectified in Fig. 1b by moving the initialization statements inside the try block. Furthermore, while the buggy and clean code files shown in Fig. 1c,d, respectively, share identical static features like code complexity and size, they differ in their semantic structures. Specifically, Fig. 1c lacks a loop increment statement within its do-while blocks, leading to an infinite loop bug. This distinction highlights the importance of semantic analysis in identifying defects that traditional static features might overlook.

The Java code files in the previous example share identical characteristics regarding code size, function calls, and complexity, among other source code properties. Relying on traditional features such as code complexity and size to characterize these two Java files would result in indistinguishable feature vectors. Nonetheless, there is a profound difference in the semantic information between these two lines of code. Therefore, incorporating semantic information becomes essential for developing higher-accuracy prediction models, as it captures nuances that traditional metrics cannot. In contrast, semantic features such as the complexity feature cannot present statistical details of the program. Using only semantic features will cause this statistical information loss and negatively affect prediction accuracy.

To bridge the divide between semantic and traditional features in SDP, we propose a SDP model that integrates and leverages both types of features. Our proposed model is a hybrid CNN-MLP classifier; semantic information extracted from AST is fed into a CNN, and traditional features extracted from the PROMISE repository are fed into MLP. The proposed model also provides a gated merging mechanism, which fuses the outputs of CNN and MLP to determine the merging ratio of both features.

Deep learning techniques, especially CNN, have succeeded remarkably across various domains, including visual pattern classification^14–16^, natural language processing^17,18^, and computer vision^19–21^. CNNs excel at learning and extracting complex patterns and features from data, which is particularly beneficial for capturing semantic information from source code. This capability enhances the model’s ability to detect subtle defects that traditional methods might overlook, leading to more accurate and reliable defect prediction. In this work, we investigate the CNN-MLP performance based on both traditional and semantic features by applying various measures under various evaluation cases. The performance evaluation of the proposed approach is conducted using widely adopted evaluation metrics in the field of defect prediction research, including the F1, AUC score, and the PofB20 measure. The results of experiments on seven projects prove that the proposed CNN-MLP model outperforms the other baseline techniques in terms of F1 and AUC under non-effort-aware scenarios. In addition, under an effort-aware condition, CNN-MLP outperforms the other baseline techniques in terms of PofB20.

This paper produces the following contributions:

- Proposes a new method to leverage both kinds of features (traditional and semantic) using a hybrid CNN-MLP model, where the CNN model is fed with semantic features extracted from source code, and the MLP model is provided by traditional features extracted from the PROMISE dataset.
- Conducts large-scale experiments to assess the performance of the CNN-MLP model for defect prediction activities by fusing traditional and semantic features.
- Evaluate the proposed method’s performance through a comparative analysis with seven established techniques: TR, CNN, DBN, LSTM, DP-HNN, SDP-BB, and ACGDP. This evaluation aims to elucidate our approach’s strengths and potential improvements in relation to these well-regarded methodologies.

The remainder of this work is summarized as follows: In Section “Related work”, we review the related work. The methodology of the proposed framework is described in Section “Methodology”. Section “Experimental settings” discusses empirical studies and experimental settings. Section “Results analysis and discussion” provides and discusses the experimental results obtained from the study. The paper concludes with Section “Conclusions and future work”, which summarizes the key findings and future works.

## Related work

### Traditional software defect prediction

Source Code Defect Prediction has garnered significant interest within the field of software engineering. SDP models provide a prioritized list of software components that are likely to contain defects, thus allowing quality assurance teams to allocate their finite investigative efforts and testing resources more effectively^22,23^. Previous research has introduced various SDP approaches that leverage traditional features; Wu et al.^24^ suggested a semisupervised dictionary learning approach that uses many unlabeled data and labeled defect data in a small amount. They conducted their experiments on the metrics of NASA Dataset^25^, such as code size, readability, complexity, etc. Furthermore, their approach incorporates the cost of classification mistakes into the process of dictionary learning. Li et al.^26^ applied active learning based on the semi-supervised technique to build a samples-based prediction model. They performed the experiments and evaluated the results on static metrics from PROMISE datasets^27^, including Depth of Inheritance Tree, Average Cyclomatic Complexity, Number of Dependent Classes, etc. Abaei et al.^28^ introduced a hybrid SDP model based on an artificial neural network and a self-organizing map. This model was developed to identify the defects in a semi-supervised method using software features threshold values without high-quality data. They used the NASA MDP metrics to perform their experiments. Wang et al.^29^ introduced a semi-boost algorithm that uncovers hidden clustering relationships among modules within an enhancement framework. They assessed their model’s effectiveness using software metrics from the NASA Dataset. On another front, Zhang et al.^30^ developed a method to create a balanced training dataset for classifying fault-free programs, utilizing Laplacian point sampling. They devised a technique to assign weights to a relationship graph through a non-negative sparse algorithm. The proposed method employs label propagation algorithms to identify the identities of unlabeled packages, relying on a specially constructed non-negative sparse graph. To optimize their strategy for handling unlabeled data, they experimented with adjusting the proportion of labeled software modules between 10 to 30% within the NASA datasets, exploring the impact of varying levels of labeled data on the prediction accuracy.

Zhu et al.^31^ unveiled a novel defect prediction strategy utilizing the Naive Bayesian algorithm. Their method crafts a training model by considering data gravity and the weight of feature dimensions. Subsequently, it employs these information weights to determine the prior probabilities of the training data, thereby establishing a predictive classifier specifically designed for source code defect prediction. Their experiments were conducted on the defect change metrics proposed in Kamei Dataset^32^. Through domain adaptation, Jin^1^ employed kernel-twin support vector machines to adapt to different training data distributions. The author used KTSVMs with domain adaptation functions as a cross-project defect prediction (CPDP) model in this study. This study conducted experiments on both software metrics from Kamei and PROMISE datasets. Ryu et al.^33^ studied whether class imbalance training can assist with CPDP models. The similarity weights and cost of asymmetric misclassification generated from distribution features are strongly linked in their approach to determining the optimal resampling strategy. He et al.^34^ studied the feasibility of CPDP, concentrating on training data selection and defect prediction in a cross-project scenario.

### Software defect prediction based on semantic features

While numerous traditional methods have been developed for Source Code Defect Prediction (SDP), their predictive accuracy remains in scope for enhancement. Integrating semantic features into SDP models is a growing trend that has shown promising results in improving prediction outcomes^35,36^. In an innovative approach, Wang et al.^10^ utilized DBN for defect prediction, leveraging semantic information extracted from the source code. Their model employs a DBN architecture designed to decode semantic descriptions within software projects, utilizing both the changes in source code and the abstract syntax trees (ASTs) of programs as inputs for predictions at the change level and file level.

Expanding on this concept, Fan et al.^37^ presents an SDP framework that employs an Attention Recurrent Neural Network (ARNN). This advanced framework converts AST nodes derived from the source code into high-dimensional integer vectors. These vectors are then processed as inputs for the ARNN, utilizing dictionary mapping and word embedding techniques to facilitate this transformation. Further extending the work of Wang et al. ^10^, Wang et al.^38^ enhanced the existing model by incorporating semantic features extracted from the programs’ ASTs. Their approach focuses on defect prediction in two specific contexts: file-level and source code changes. By applying these semantic features, the extended model offers a more nuanced and effective method for predicting software defects, showcasing the evolving landscape of SDP methodologies and the increasing significance of semantic analysis in this field.

Majd et al.^13^ introduced an LSTM network approach to construct an SDP model that leverages statement-level code metrics. They outlined two categories of statement-level features designed to assess various aspects of code statements. The first category, external-linear metrics, focuses on gathering contextual information surrounding a statement that could influence its complexity. The second category, internal-linear metrics, aims to ascertain the complexity of the statement itself based on its intrinsic characteristics. This dual approach allows for a comprehensive evaluation of statement complexity, facilitating more accurate defect prediction at the statement level. Deng et al.^39^ employed an LSTM network to derive semantic metrics from source files. By analyzing each file’s AST and processing it through an LSTM, they extracted semantic metrics of the program. These metrics were subsequently utilized to detect faults within the file, showcasing the effectiveness of LSTM in understanding and leveraging the semantic aspects of source code for defect identification. Dam et al.^40^ proposed an SDP model based on a tree-LSTM. The proposed model used an LSTM network that perfectly matches the AST source code representation. Shi et al.^41^ also proposed a model for defect prediction using a Bi-LSTM network and AST path pair representation.

Other methods have also been proposed for SDP based on semantic features; Liang et al.^42^ proposed a framework to represent a semantic feature based on word embedding. They used the CBOW model to conduct token embedding and LSTM to predict defects. Li et al.^43^ proposed an SDP model based on CNN. Their approach based on the programs’ ASTs; token vectors are extracted and encoded as numerical values. Firstly, CNN learns semantic information from programs through these numerical vectors. After that, the semantic information are used for defect prediction. Meilong et al.^44^ presented a CNN model to learn source code semantic features and then use these semantic features for predicting defects. Transformer models have been applied to source code representation and software defect prediction. Guo et al.^45^ introduced GraphCodeBERT, a multilayer transformer architecture, which takes three primary elements as its input: the source code, data flow graph, and accompanying comments. This framework facilitates a range of code-centric tasks, including code translation, code refactoring, code clone detection, and other operations centered around source code analysis and manipulation.

Huang et al. ^46^ proposed an SDP model based on an attention mechanism. Their model leverages the code semantics as an entry point by leveraging AST representation and introduces a mask model to establish connections between the function methods across the project files. Yao et al. ^47^ proposed a new approach for SDP. Their method focused on extracting semantic information from the code’s text structure and then utilizing this information to identify software defects. The idea of their approach lies in its ability to mine semantic information from software, which enhances the accuracy of SDP. Uddin et al. ^48^ introduced a new SDP approach using Bidirectional LSTM (BiLSTM) and BERT to extract semantic information from code files. Their model enhanced the accuracy of software defect predictions by effectively capturing the code’s semantic information. These advanced techniques significantly improved over traditional defect prediction methods, showcasing better performance in identifying potential software defects. Siki’c et al.^49^ presented an approach for SDP using a Graph CNN (GCNN). They first extracted the AST from code files and then leveraged the graph representation of ASTs to capture detailed syntactical information. The GCNN model demonstrated better performance in identifying defects in software code over traditional methods, as it effectively processes the complex relationships and patterns inherent in source code syntax. Yu et al.^50^ proposed an AST-based representation method for file-level defect prediction, enhancing fine-grained detail and long-term dependency detection. They also introduced the DP-HNN framework to extract key features from AST’s hierarchical structure and predict defects in Java files. Qiu et al.^51^ developed a tree-based encoding strategy utilizing hybrid granularity levels for predicting defects. Specifically, they enhanced defect prediction accuracy by introducing five granular selection approaches for generating varied ASTs from the code. Subsequently, they utilized a tree-based continuous bag-of-words model to convert AST nodes into numerical vector formats, maintaining the hierarchical structure inherent to the code.

Some works have attempted to combine software features through simple concatenation to enhance performance. Ni et al.^52^ introduced a just-in-time (JIT) defect prediction approach that integrates semantic features extracted from source code with process features related to the software development process. To evaluate the feasibility of their JIT-Fine approach, they created a large-scale, line-level dataset called JIT-Defects4J, which was manually labeled with defect information. Li et al.^43^ introduced an SDP model that leverages CNNs. Their approach operates on the programs’ ASTs. It involves extracting token vectors from the ASTs and encoding them as numerical representations. The proposed model amalgamates the semantic features extracted by the CNN from the code structure with traditional software metrics through simple concatenation to enhance the overall predictive performance of the defect prediction model. Qiu et al.^53^ proposed a CPDP model that utilizes deep learning techniques to extract features from the AST using a CNN. Furthermore, the model learns transferable joint features by integrating deep learning-extracted and handcrafted features, achieved through applying a transfer component analysis algorithm.

Unlike their traditional approaches that simply concatenate semantic features with traditional features, our model employs a hybrid CNN-MLP classifier to extract and fuse semantic and traditional features efficiently. It then leverages a fully connected gate for fusing features and effectively combining the extracted features from different sources. By integrating a CNN with an MLP, our hybrid CNN-MLP model can capture not only traditional features but also the inherent semantic nuances present in software project source code. This ability to comprehend and process both traditional and semantic features gives our model a distinct advantage, enabling it to discern patterns and make classifications with higher accuracy and confidence. The success of CNN-MLP lies in its hybrid architecture, which merges two powerful machine-learning paradigms. The CNN components excel at processing data with grid-like topology, such as spatial structures, making them adept at identifying patterns in the arrangement of code elements. Meanwhile, MLPs are proficient at handling the abstract features extracted by CNN, allowing for a more nuanced understanding of the source code. This synergy between CNNs and MLPs facilitates a comprehensive feature extraction process, crucial for tasks that require an in-depth understanding of the data, as is often the case in software engineering.

## Methodology

As mentioned in the previous section, the traditional features cannot catch the different semantic information of programs. In contrast, semantic features often cannot represent contextual information of the code file, such as the program complexity feature. Only focusing on one kind of these features degrades the efficiency of the prediction models.

**Figure 2:**
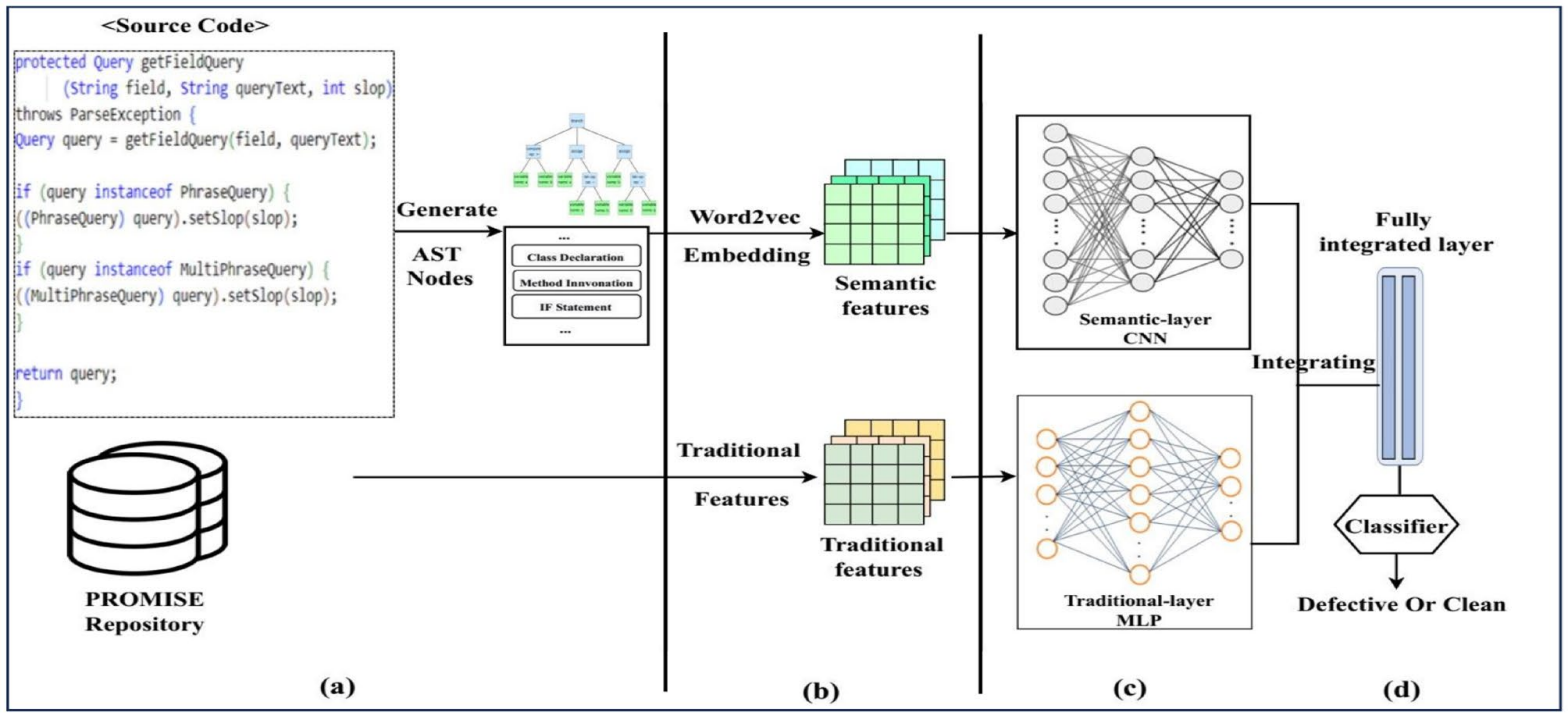
Overview of our CNN-MLP approach. (**a**) Parsing source code, (**b**) data preprocessing, (**c**) CNN-MLP layers structure, and (**d**) merge and concatenate layer.

To bridge this gap, we propose a hybrid model named CNN-MLP. Our proposed method uses both features (semantic and traditional). Fig. 2 demonstrates the whole workflow of the CNN-MLP approach.

First, we parse the source code files and generate AST nodes. Then, we select representative AST nodes and employ word2vec embedding to convert them into numeric vectors as semantic features. During data preprocessing, traditional features are also extracted from the PROMISE dataset after the source code’s semantic information is extracted. After that, we build the CNN-MLP layers structure; the semantic features will be used as input to CNN, and traditional features will be used as input to MLP. Finally, the outputs of the CNN and MLP models will be fed into a classifier layer called the fully connected layer to classify programs prone-defect (clean or buggy).

### Parsing source code

In this section, we analyze code files to produce nodes for abstract syntax trees; this process results in the creation of four distinct types of nodes: (1) nodes of class instance creation and method invocations. (2) Control-flow nodes, e.g., IFStatement, WhileStatement, ForStatement, etc. (3) Declaration nodes, including type declarations, enum declarations, and method declarations. (4) Other nodes such as Formal Parameter and Basic Type.

**Figure 3:**
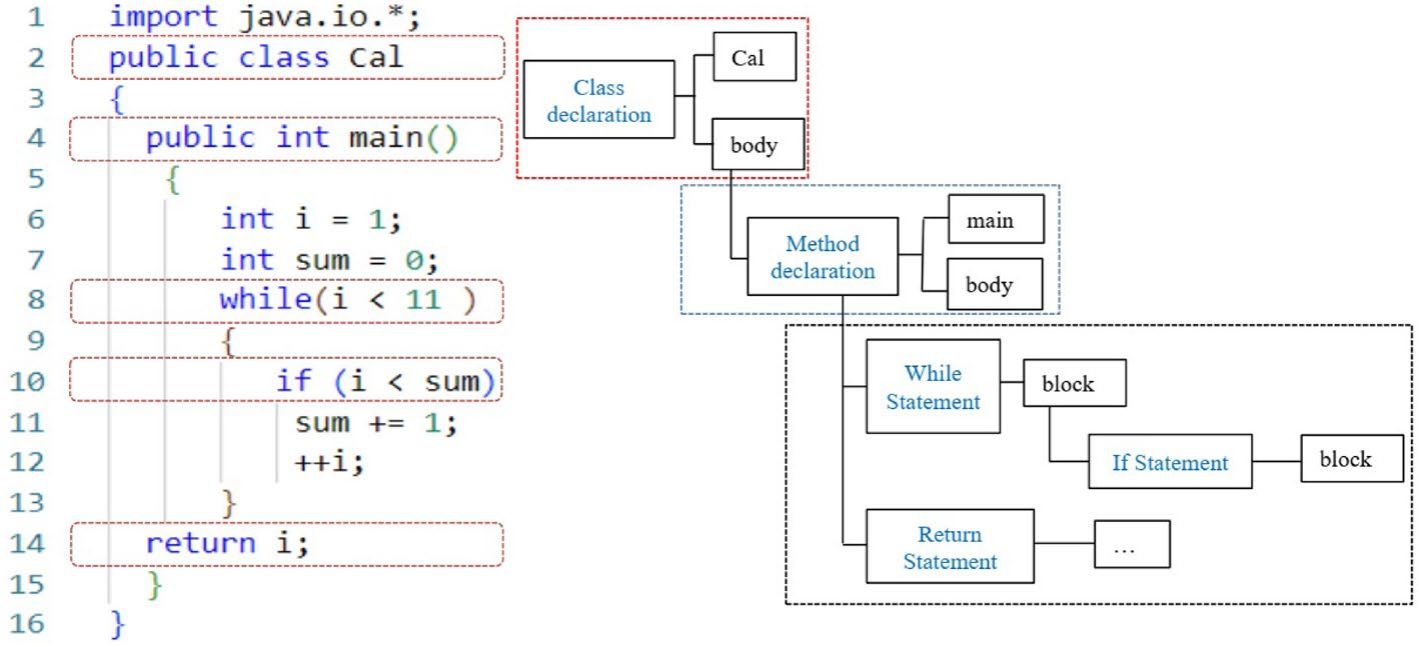
Parsing source code example.

For example, Fig. 3 represents the process of analyzing a Java source file and converting it into an AST. The resulting AST is then tokenized into a vector described as [Cal, main, while, if]. In this vector, Cal signifies an instance creation node, representing the creation of an object from the Cal class. main is a declaration node, indicating the declaration of the main method in the Java file. The while and if are control-flow nodes, representing the while-loop and if-statement, respectively. This tokenization captures the essential structural elements of the source file, providing a compact representation of its syntactic components. It’s important to note that the tokenization process is typically the first step in constructing an AST from the source code. The tokens are then organized into a hierarchical tree structure that represents the code’s syntactic structure, with different node types corresponding to the various language constructs, such as instance creation, declarations, and control-flow statements.

### CNN-MLP structure

**Figure 4:**
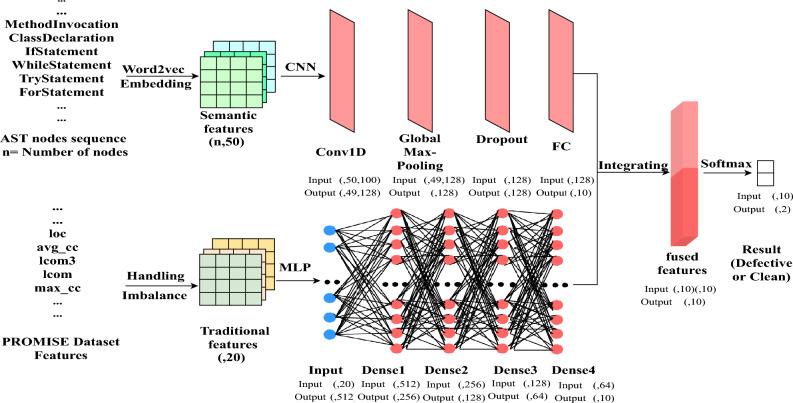
Layers structure of CNN-MLP.

This study proposes a hybrid model combining CNN and MLP to synergistically leverage traditional software metrics and semantic features extracted from source code. The architecture of the proposed CNN-MLP model integrates elements of both CNN and MLP; the structural layers of this hybrid model are depicted in Fig. 4.

#### CNN layers structure

As illustrated in Fig. 4, the implemented CNN model consists of several key components: an input layer, a one-dimensional convolutional layer (Conv1D), a global max-pooling layer, a hidden dropout layer, and a fully connected layer. The activation function employed across all CNN layers is ReLU. This configuration harnesses CNN’s robust ability to generate features and extract the semantic content from source code.

#### MLP layers structure

As shown in Fig. 4, our proposed MLP contains five layers: one input layer and four dense layers. The input layer consists of 20 neurons and receives the traditional features. Four dense layers connect each input with every merged layer using weights. All dense layers except the last layer use RELU activation, and the last dense layer uses Sigmoid.

### Gated merge layer

In this phase, the output of CNN and the output of MLP are fed into this merging gate separately, where the information passing through is filtered using a fully integrated layer. The outputs of the CNN and the MLP are then integrated according to the following:

1 $$\begin{aligned} G_{m}= \text{ integrate } \left( \left( \sigma \left( W_{t} \cdot M_{t}\right) +l_{t}\right) , \left( \sigma \left( W_{s} \cdot C_{s}\right) +l_{s}\right) \right) \end{aligned}$$

In this formulation, $M_{t}$ denotes the MLP model’s final output, with $W_{t}$ signifying the MLP model’s weights. Similarly, $C_{s}$ is the CNN model’s final output, while $W_{s}$ stands for the CNN model’s weights. The terms $l_{s}$ and $l_{t}$ are the learning parameters for the gates, and $\sigma$ symbolizes the sigmoid function.

In this phase, a softmax layer processes the output from the gated merge layer (Gm output) and determines whether the program contains bugs, which is defined by:

2 $$\begin{aligned} y={\text {softmax}}\left( W \cdot G_{m}+b\right) \end{aligned}$$

where W represents the weights of the softmax layer, and b refers to the bias term.

## Experimental settings

### Dataset description

In this work, we choose various open-source projects from the PROMISE repository (https://openscience.us/repo/defect/), where the description of the selected projects is provided in Table 1. All projects in the PROMISE dataset used in this study include CSV data and Java source code files. The first column in the CSV files contains the path to the Java code. We utilize these paths to read and extract the AST from the Java files. We chose these seven projects to cover several kinds of data types, classes, functions, control flow, etc. PROMISE dataset has been utilized extensively in several studies to create practical SDP models^12,38,43,54,55^. We chose the PROMISE dataset because of its wide use, which indirectly compares the proposed approach with previous research.

**Table 1:** Description of dataset projects.

Project	Versions	Description	Avg files	Defects rate (%)
xalan	2.4, 2.6	A Java library for processing XML files.	804	32.3
poi	1.5, 3.0	Java library for accessing Microsoft files.	340	62.1
ant	1.7	A Java-based build code files.	745	22.2
log4j	1.1	A Java-based logging library	109	33.8
jEdit	4.0, 4.1	A text editor built for programmers.	309	24.9
lucene	2.0, 2.2	An open-source text search library.	221	52.9
synapse	1.1, 1.2	Adapters for transmitting data	239	30.5

First, we use each project’s version numbers since we need the archive of its source code to extract AST nodes from the program and then generate the integer vectors from these nodes to feed our CNN model. In this work, we extracted four categories of AST nodes: Method declarations and invocations, Class instance creations, control flow, and other nodes such as Formal Parameter, Basic Type, Member Reference, etc. Table 2 shows the details of these categories. After that, we use Word2vec embedding to generate the token sequences from the extracted AST nodes and then use these token sequences to create semantic features for training the CNN model as described in Section “CNN-MLP structure”.

**Table 2:** AST Nodes Categories.

Categories	AST Nodes	Notes
Class instance creations/ Method invocations	Method Invocation Super Method Invocation Object creation Recursive function	Methods and Class instances are recorded as explicit text in the source code. Each contains only one statement of a method invocation or instance creation.
Declarations	Method Declaration Class Declaration Constructor Declaration Variable Declaration Package Declaration Interface Declaration	Besides the invocation, the declaration of AST node types are more valuable since they can present additional semantic information.
Control flow	If Statement Do Statement For Statement Break Statement Enhanced For Statement Continue Statement While Statement Try Statement Return Statement Throw Statement Switch Statement Switch Statement Case Block Statement Statement Expression Try Resource Catch Clause Catch Clause Parameter	Although it is essential to recognize the AST invocation and declaration nodes, each method declaration node only has one method invocation node. As a result, a representation with only one request within is unlikely to be buggy in all instances. Compared to utilizing method names, employing the AST node types control flow provides more useful semantic information.
Others	Formal Parameter Basic Type Member Reference Super Member Reference Reference Type Assert Statement Synchronized Statement	Node types are used to record control-flow nodes. In addition, the nodes of Member Reference, Assertion Statement, etc., are kept as their values.

Our second baseline of dataset description is to handle the traditional features; the traditional features we considered include 20 traditional metrics. Table 3 lists the details of these features. On the PROMISE data, we applied the imbalanced data handling approach outlined in Section  “Data preprocessing”.

**Table 3:** PROMISE traditional dataset features.

Feature	Description	Feature	Description
DIT	Depth of Inheritance Tree	DAM	Data Access Metric
WMC	Weighted Methods per Class	NPM	Number of Public Methods
NOC	Number of Children	MOA	Measure of Aggregation
CBO	Coupling Between Object classes	MFA	Measure of Functional Abstraction
LCOM	Lack of Cohesion in Methods	IC	Inheritance Coupling
RFC	Response for a Class	CAM	Cohesion Among Methods of Class
LCOM3	Lack of Cohesion in Methods, different from LCOM	CBM	Coupling Between Methods
Avg_CC	Arithmetic mean of the CC value in the investigated class	CA	Afferent Couplings
Max _CC	Maximum value of CC methods of the investigated class	AMC	Average Method Complexity
LOC	Lines of Code	CE	Efferent Couplings

### Data preprocessing

#### Word embedding (Word2vec)

CNN is designed to process inputs as numerical vectors, with a prerequisite that these input vectors maintain uniform lengths. An initial step in incorporating semantic features into CNN involves establishing a correlation between tokens (semantic units in the source code) and integers, effectively transforming token vectors into corresponding integer vectors. Each distinct token is assigned a unique integer identifier to facilitate this conversion. However, the challenge of varying lengths in these integer vectors persists, necessitating a solution to standardize input size. The Word2vec technique is employed to address this issue and ensure the compatibility of semantic features with CNN’s input requirements. Word2vec^56,57^ plays a pivotal role in creating a consistent mapping between tokens and integers. This mapping technique overcomes the obstacle of disparate vector lengths and generates fixed-size numerical inputs suitable for CNN processing. By leveraging Word2vec, it becomes feasible to convert the rich semantic information embedded within the source code into a standardized numerical format, thus enabling the effective application of CNN for tasks that require the analysis of semantic features.

Word2vec represents a method for word embedding within the realm of Natural Language Processing (NLP), facilitating the conversion of words into computationally manageable and systematically organized vectors. It offers two distinct approaches for constructing word embeddings: Continuous Bag-of-Words (CBOW) and Skip-Gram. This study uses the CBOW model to derive integer vectors from token vectors. The design of the CBOW model is centered on predicting the target word based on the context provided by surrounding words. This approach effectively encapsulates semantic information within numerical vectors, laying the groundwork for further processing and analysis.

**Figure 5:**
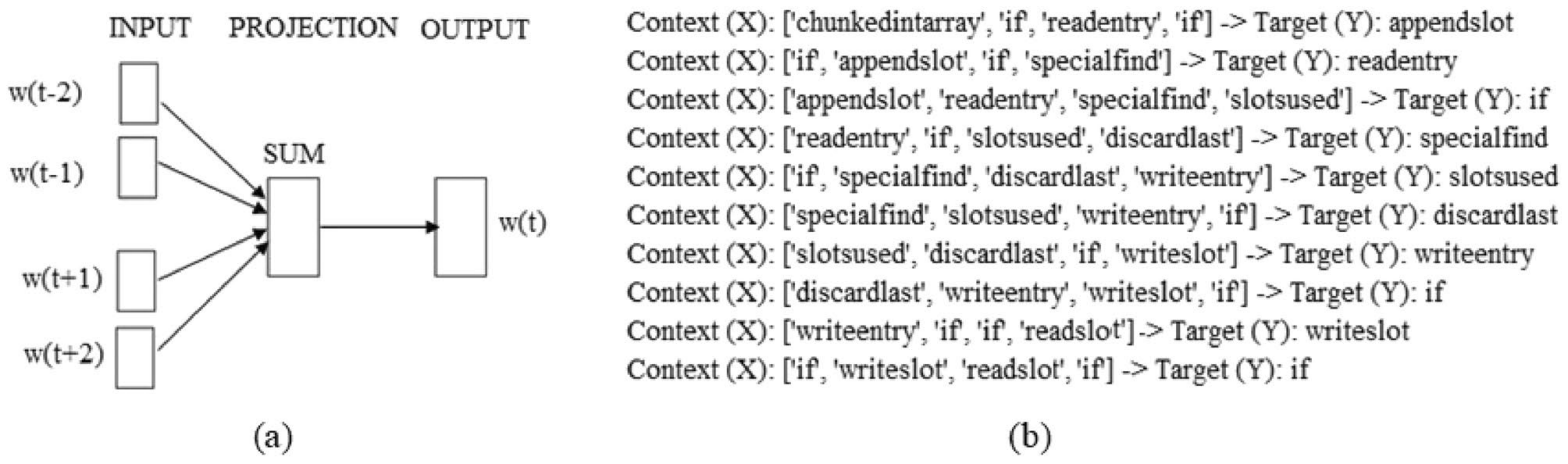
Continuous bag-of-word example. (**a**) The CBOW model architecture and (**b**) the CBOW (context, target) example.

Figure 5 shows the CBOW example; Fig. 5a presents the general mechanism of CBOW, and Fig. 5b presents the detailed steps of COW. As depicted in Fig. 5b, in analyzing token vectors, such as “ChunkedIntArray if appendSlot readEntry if specialFind slotsUsed discardLast writeEntry if writeSlot if readSlot if,” these can function either within a context window or as target words. As depicted in Fig. 5b, using a context window of size 4 enables the model to predict the target word based on the surrounding context words. This predictive mechanism is fundamental to how the word2vec model operates, leveraging the immediate linguistic environment to understand and predict word usage. In this particular study, we have configured the word2vec model to utilize a vector size of 100 and a context window size of 5. This configuration is chosen to optimally balance the granularity of semantic representation with computational efficiency, allowing for a nuanced capture of semantic relationships within a manageable computational framework. By adjusting these parameters, we aim to improve the model’s performance to accurately model linguistic patterns and relationships, thereby improving the overall effectiveness of the semantic feature extraction process.

#### Handling imbalance

Datasets of SDP frequently exhibit class imbalance, where buggy instances constitute only a minor fraction of the total dataset. This imbalance ratio varies, directly correlating to the defect rate within the dataset. For instance, among the projects detailed in Table 1, the ’ant’ project displays the most significant imbalance, showcasing a buggy rate of 22.2%. Such imbalance poses challenges to model performance, particularly affecting its proficiency in accurately identifying non-defective instances, as highlighted by comprehensive studies in the field^58,59^.

Addressing the issues arising from imbalanced data is crucial for improving model accuracy and reliability. As detailed by^60,61^, two prevalent strategies for mitigating these challenges include Oversampling and Undersampling. Oversampling is the process of duplicating instances from the minority class (defective files) to achieve a balanced dataset representation while undersampling involves reducing the number of instances from the majority class (non-defective files).

In this study, we opt for the Undersampling approach. This preference is guided by the rationale that Undersampling maintains the integrity of the original dataset by using only genuine instances, thus avoiding the potential introduction of artificial bias that might occur with Oversampling. This approach ensures that our training sets accurately reflect real-world conditions, providing a more reliable basis for model training and evaluation. By prioritizing authenticity in our dataset composition, we aim to enhance the model’s predictive performance in a practical and effective manner for software defect prediction tasks.

### Parameters setting

We divide our experiments into two main implementation steps: Semantic features extraction and defect classification. In the first phase, to generate the semantic features, we use Word2vec in deeplearning4j3 to construct a group of word embedding by changing the size of the context window and the dimensionality. We leverage the studies of^57,62,63^ to set values for the context window sizes, dimension size, batch size, negative sampling, minimum word frequency, and iterations. Table 4 shows the details of these parameters. In the second phase, the parameters for the classifier model are defined; we assign values for the group of parameters like the number of input layers, hidden layers, and nodes in each layer. We additionally consider batch size, epoch, the activation functions used in the input and hidden layers (CNN-MLP activation), fully connected activation, Merged activation function, optimizer, and learning rate. Table 4 shows the details of these parameters. We build our CNN-MLP model using python-3.9.6 with Tensorflow-2.5.0 and Keras-2.5.0. Other implementations are executed on Gensim for word2vec embedding, Pandas for processing dataset, and Javalang and NLTK for generating AST. The code was run on CPU Intel®Core™ i7 with NVIDIA GeForce MX250 CUDA 11.2.

According to our findings in previous studies^38,64^, validation techniques like k-fold cross-validation often introduce significant bias when evaluating SDP models, leading to inaccurate assessments. In this work, we combined two features (semantic and traditional). We performed several procedures on the dataset, including processing imbalanced data and integrating semantic features with standard features. We did not use it in this study to avoid the issues associated with k-fold cross-validation. Instead, we evaluated the performance of our CNN-MLP model by building a prediction model using data from different releases (see Table 1). We also employed various performance measures to assess the model’s performance in non-effort-aware and effort-aware scenarios.

In this study, we employed several baseline methods and compared their performance against our proposed model, as described in Section “Baseline methods”. The hyperparameters for each technique were carefully tuned based on the recommendations from their respective literature. Specifically, for the CNN model, we utilized 10 hidden layers, each comprising 100 nodes. Additionally, we set the number of filters to 10 and the filter length to 5. The DBN model consisted of 10 hidden layers, with 100 nodes in each layer. The LSTM model was configured with 16 LSTM units per layer, 250 attention widths, and a vector dimension of 16 for calculating the attention widths. The DP-HNN model had 5 hidden layers, each containing 100 nodes. The AdaMax optimizer was employed with a default learning rate of 0.002. We utilized two Bidirectional LSTM (BiLSTM) layers for the SDP-BB model, each comprising 128 units. Furthermore, seven hidden layers with hidden sizes of 8, 16, 32, 48, 64, 128, and 256 were incorporated. The Adam optimizer was used with a fixed learning rate of 0.001. The ACGDP model consisted of 5 layers, with a hidden size 249 and a dropout rate of 0.361.

To ensure a fair comparison, the number of epochs was set to 100 for all models, including our proposed method. This consistent epoch setting allowed for a comprehensive evaluation of the models’ performance under identical training conditions.

**Table 4:** Parameters of the proposed approach.

Type	Parameter	Value
Extraction	Context window sizes	5
	Vector size	100
	Batch words	50
	Negative sampling	10
	Minimum word frequency	5
Classification	CNN-MLP layers (input and hidden).	10
	Batch size	1024
	Epoch	100
	CNN-MLP activation.	RELU
	Fully connected activation	Sigmoid
	Merged activation	Softmax
	Optimizer.	Adam
	Learning rate	Default (0.01)

### Baseline methods

This section introduces the baseline methodologies utilized in our study. To ascertain the efficacy of our newly proposed model, we have chosen seven distinct methods to serve as our comparative baselines. These baseline models incorporate various features-spanning traditional metrics, semantic attributes, simple integrating approaches, and changes in source code-paired with a diverse array of classifiers ranging from conventional machine learning algorithms to more advanced deep learning frameworks. This selection is designed to provide a comprehensive benchmark, allowing us to thoroughly evaluate the performance of our proposed model against established methods that utilize different combinations of features and classification techniques. By doing so, we aim to highlight our model’s unique strengths and potential advantages in accurately predicting outcomes based on the analyzed features.

#### Traditional (TR)^65^

TR is a method that uses 20 traditional handcrafted code metrics shown in Table 3 as input to train a classifier (Naïve Bayes and Random Forest).

#### CNN^43^

CNN serves as a predictive model for detecting software defects. It utilizes ASTs as input data to identify semantic elements within the source code. This approach amalgamates the semantic features extracted by CNN with traditional software metrics through simple concatenation, aiming to enhance the overall predictive performance.

#### DBN^38^

A defect prediction model that utilizes semantic features and features derived from source code changes, created using a Deep Belief Network.

#### LSTM^39^

An SDP framework leverages LSTM networks to extract syntactic features directly from program file ASTs. These extracted syntactic features are then utilized as inputs to predict the presence of software defects within the codebase.

#### SDP-BB^48^

An SDP approach using BiLSTM and BERT to predict defects in software code effectively. This model enhances defect prediction accuracy by leveraging two deep learning models to understand the semantic features of code.

#### DP-HNN^50^

A defect prediction framework based on the Hierarchical Neural Network. This model capitalizes on the hierarchical nature of ASTs, strategically segmenting extensive file-level ASTs into multiple subtrees centered around key AST nodes pivotal to the SDP task.

#### ACGDP^66^

An Augmented-Code Graph Defect Prediction model that extracts features from the code’s graph representation. Subsequently, graph neural networks are applied to these extracted features to capture intricate patterns and make predictions regarding defects within software modules.

#### CNN-MLP

It is the prediction model introduced in this study.

For the experiment’s integrity and to validate the outcomes, we implement undersampling as described in  4.2 to address imbalances in the dataset, employing it as our chosen technique for balanced learning. Each experiment is conducted 30 times to ensure reliability and consistency in the results.

### Performance measures

This study evaluates the proposed approach performance under non-effort-aware and effort-aware scenarios.

#### Non-effort-aware evaluation measures

In this scenario, it is presumed that sufficient resources are available to facilitate testing based on the outcomes of the defect prediction model, meaning that every predicted defective instance can undergo verification. SDP models determine the outcome of a code modification through four possible predictions: (1) correctly identifying a defective code change as defective (True Positive, TP), (2) inaccurately identifying a defective code change as non-defective (False Negative, FN), (3) correctly identifying a non-defective code change as non-defective (True Negative, TN), and (4) inaccurately identifying a non-defective code change as defective (False Positive, FP).

Given these four outcomes, the predictive model computes key performance metrics within the test dataset, including recall, F1 scores, and precision. In this study, we have selected F1 and AUC as the performance indicators to demonstrate the efficacy of our approach under conditions that do not take effort into account.

The following are the detailed definitions:

*Recall* refers to the ratio of all correctly classified faults to all faults.

3 $$\begin{aligned} \text{ Recall } =\frac{T P}{T P+F N}. \end{aligned}$$

*Precision* is defined as the proportion of fault changes correctly identified relative to the total number of fault classifications made incorrectly, which is given as

4 $$\begin{aligned} \text{ Precision } =\frac{T P}{T P+F P}. \end{aligned}$$

*F1 scores* An integrated metric that merges both recall and precision rates, representing the harmonic mean of precision and recall, which is defined as

5 $$\begin{aligned} F1=\frac{2 \times \text{ precision } \times \text{ recall } }{ \text{ precision } + \text{ recall } }. \end{aligned}$$

*AUC* The Area Under the Curve (AUC) of the Receiver Operating Characteristic (ROC) is a critical evaluation metric used in the field of real-time Source Code Defect Prediction (SDP) research. When assessing the performance of a model classifier, the ROC curve is constructed by setting various classification thresholds. The x-axis (abscissa) of the ROC curve denotes the false positive rate (FP rate), while the y-axis (ordinate) represents the true positive rate (TP rate). The ROC curve is composed of the coordinate points derived from each classification threshold’s pair of FP and TP rates. The AUC, the area beneath the ROC curve, varies from 0 to 1, with higher values indicating better model performance.

#### Effort-aware evaluation measures

Effort-aware conditions are implemented when testing resources are constrained or deadlines are imminent, representing the typical context in which defect prediction techniques are applied in real-world scenarios. Under such conditions, only a limited number of the predicted defect instances can be examined. Hence, in effort-aware scenarios, assessing the predictive performance using measures specifically tailored to these circumstances is essential. In this study, we utilize the PofB20 metric as the evaluation criterion for the effort-aware condition.

*PofB20*^67^ is a measure designed to quantify the proportion of defects a programmer can identify by examining 20% of the Lines of Code (LOC). This metric becomes applicable once the programmer has inspected 20% of the LOC within the test dataset. At this juncture, the PofB20 scores are expressed as the percentage of faults uncovered due to the inspection process. The possible range for PofB20 values lies between 0 and 1, where a higher value signifies a more efficient model performance. Essentially, this metric offers a focused lens on the model’s capability to prioritize and reveal the most significant defects early in the inspection process, thus serving as an essential indicator of the model’s practical utility in streamlining defect detection efforts under constrained conditions.

To calculate the PofB20 metric, we initiate the process by arranging the instances within the test files in descending order according to their confidence levels-the model’s assessed probabilities that each instance is likely defective. A higher confidence level suggests a greater likelihood of an instance being defective. Subsequently, we tally both the lines of code that have been scrutinized and the defects that have been uncovered in the process. The inspection halts once 20% of the Lines of Code (LOC) in the test dataset have been reviewed. At this point, the proportion of detected defects relative to this 20% examination is recorded as the PofB20 score. Essentially, a superior PofB20 score signifies the model’s enhanced efficiency in uncovering a larger number of bugs by examining a constrained segment of the LOC, highlighting the model’s effectiveness in prioritizing code segments that are most probable to contain defects.

## Results analysis and discussion

The following research questions steered the conducted experiments and the analysis of their outcomes:

RQ1: How does our proposed CNN-MLP model outperform state-of-the-art models under non-effort-aware scenarios?
RQ2: How does our proposed CNN-MLP model outperform the state-of-the-art models under effort-aware scenarios?
RQ3: How long does it take to train the CNN-MLP model, and how long does it take to generate the semantic features?

*For RQ1#*, in Table 5, we observe the F1 scores for both CNN-MLP and seven state-of-the-art methods (TR, CNN, DBN, LSTM, DP-HNN, SDP-BB, and ACGDP). The presentation of the highest scores in bold highlights the superior performance in each metric across the eight methods.

To delve deeper into the analysis, 12 sets of experiments were conducted, each corresponding to a specific project version number, as indicated by labels like “Xalan-2.4.” These experiments utilize source codes from respective project versions to ensure a comprehensive evaluation.

The results presented in Table 5 indicate that CNN-MLP consistently outperforms all other models with an average F1 score of 0.703. This represents a notable improvement over the next best model (ACGDP), which has an average F1 score of 0.668. Such results underscore the efficiency of combining CNNs with MLPs in handling the text and structural data inherent in software engineering tasks.

For instance, in the Poi-1.5 project, CNN-MLP achieved an F1 score of 0.856, surpassing the SDP-BB score of 0.817. This significant improvement can be attributed to the CNN-MLP model’s ability to better capture the contextual and temporal dependencies within the data. Similarly, in the log4j-1.1 project, the CNN-MLP model achieved an F1 score of 0.845, substantially higher than that of DP-HNN, which scored 0.733.

**Table 5:** F1 Values of TR, CNN, DBN, LSTM, DP-HNN, SDP-BB, ACGDP, and CNN-MLP.

Projects	TR	CNN	DBN	LSTM	DP-HNN	SDP-BB	ACGDP	CNN-MLP
jEdit-4.0	0.523	0.620	0.623	0.616	0.622	0.651	0.630	**0.675**
jEdit-4.1	0.389	0.634	0.477	0.593	0.630	0.627	**0.661**	0.652
Ant-1.7	0.563	0.621	0.661	0.711	**0.732**	0.715	0.704	0.719
Synapse-1.1	0.504	0.419	0.474	0.560	0.534	0.565	0.523	**0.572**
Synapse-1.2	0.610	0.654	0.563	0.688	0.681	0.673	0.636	**0.690**
Xalan-2.4	0.618	0.668	0.684	0.652	0.640	**0.728**	0.667	0.689
Xalan-2.6	0.549	0.643	0.669	0.644	0.563	0.673	0.682	**0.706**
Poi-1.5	0.724	0.688	0.748	0.732	0.791	0.817	0.816	**0.856**
Poi-3.0	0.774	**0.801**	0.773	0.700	0.721	0.700	0.792	0.745
log4j-1.1	0.622	0.585	0.498	0.694	0.733	0.661	0.704	**0.845**
Lucene-2.0	0.642	0.636	0.691	0.589	0.658	0.606	0.636	**0.704**
Lucene-2.2	0.609	0.623	**0.709**	0.552	0.527	0.523	0.562	0.578
Average	0.594	0.633	0.631	0.644	0.653	0.662	0.668	**0.703**

Overall, the comparative analysis underscores CNN-MLP as a superior approach in terms of both precision and recall, as evidenced by its consistently higher F1 scores across different project versions. This suggests that CNN-MLP holds promise as an effective methodology for the specific task evaluated in the experiments.

Turning to the AUC scores presented in Table 6, the CNN-MLP model also shows a commendable performance with an average AUC score of 0.616. This is higher than the average scores achieved by the other models, with SDP-BB coming closest at an average of 0.587. This superior performance is highlighted in projects like Xalan-2.4 and Synapse-1.2, where CNN-MLP scored 0.712 and 0.683 respectively, the highest across all models.

**Table 6:** AUC Values of TR, CNN, DBN, LSTM, DP-HNN, SDP-BB, ACGDP, and CNN-MLP.

Projects	TR	CNN	DBN	LSTM	DP-HNN	SDP-BB	ACGDP	CNN-MLP
Xalan-2.4	0.456	0.562	0.592	0.633	0.651	0.683	0.673	**0.712**
Xalan-2.6	0.512	0.535	0.552	0.570	0.635	**0.652**	0.620	0.626
Poi-1.5	0.436	0.553	0.502	0.546	0.560	0.534	0.548	**0.566**
Poi-3.0	0.532	0.492	0.473	0.462	0.583	0.573	0.597	**0.616**
Ant-1.7	0.541	0.601	0.596	0.658	0.622	0.631	0.636	**0.673**
log4j-1.1	0.476	0.583	0.565	0.572	0.540	0.558	0.570	**0.592**
jEdit-4.0	0.589	0.511	0.543	0.503	0.523	0.541	0.507	**0.552**
jEdit-4.1	0.488	0.589	0.534	0.481	0.501	0.519	**0.593**	0.582
Lucene-2.0	0.492	0.584	0.632	0.590	**0.641**	0.605	0.611	0.637
Lucene-2.2	0.514	0.572	0.579	0.522	0.566	0.524	0.572	**0.596**
Synapse-1.1	0.495	0.499	0.587	0.583	0.560	**0.595**	0.505	0.553
Synapse-1.2	0.595	0.574	0.592	0.667	0.620	0.633	0.600	**0.683**
Average	0.511	0.555	0.562	0.566	0.584	0.587	0.586	**0.616**

The AUC score is crucial as it provides an aggregate performance measure across all possible classification thresholds. The superior scores suggest that CNN-MLP not only predicts more accurate classifications but also maintains robustness across different operational thresholds, which is critical for practical deployments in software engineering environments.

Overall, the comparative analysis of AUC performance reinforces CNN-MLP as a leading methodology, highlighting its potential for applications requiring robust classification performance. The notable improvements over alternative approaches further validate the efficacy of CNN-MLP in various contexts, making it a compelling choice for classification tasks in practical settings.

The results of the recent study demonstrate the superior performance of our proposed CNN-MLP model compared to seven other state-of-the-art models (TR, CNN, DBN, LSTM, DP-HNN, SDP-BB, and ACGDP) across various software projects in terms of both F1 scores and AUC values under non-effort-aware scenarios. This performance advantage is particularly notable given that non-effort-aware scenarios often pose significant challenges in model training due to the absence of human effort data, which can be critical in tuning and refining the predictive capabilities of machine learning models.

The CNN-MLP model’s architecture facilitates deeper and more nuanced feature extraction, which is critical in non-effort-aware scenarios where explicit effort metrics are unavailable. By synthesizing features extracted through multiple layers, the model can infer subtle patterns that might indicate bugs or defects in software projects. Moreover, the CNN-MLP model consistently outperforms established models across a range of metrics and offers a robust solution to the challenges posed by non-effort-aware scenarios in software engineering. Its ability to effectively combine feature extraction and classification tasks results in higher accuracy and reliability, making it an excellent choice for deploying in diverse software development contexts where predictive accuracy is paramount.

**Figure 6:**
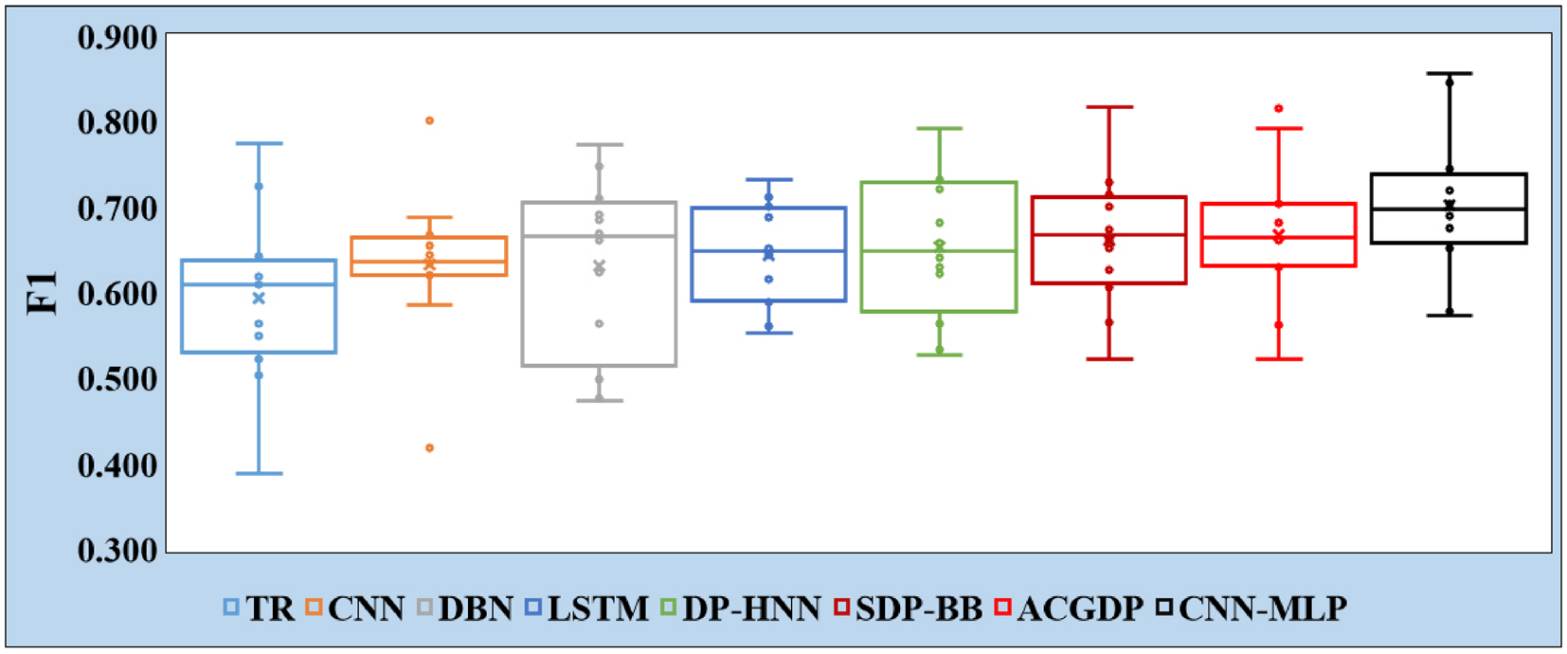
Comparison of F1 scores for TR, CNN, DBN, LSTM, DP-HNN, SDP-BB, ACGDP, and CNN-MLP.

**Figure 7:**
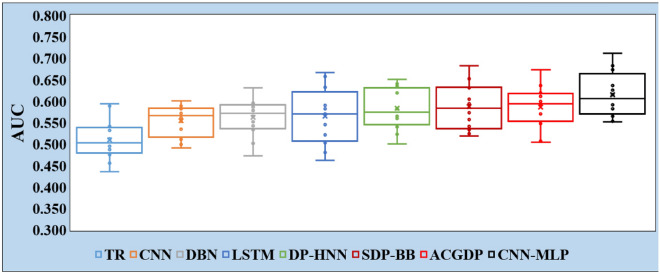
Comparison of AUC for TR, CNN, DBN, LSTM, DP-HNN, SDP-BB, ACGDP, and CNN-MLP.

In the depiction provided by Figs. 6 and 7, the boxplots serve as a visual testament to the performance of CNN-MLP compared to the baseline methods across all 12 tasks detailed in Tables 5 and 6. These boxplots, representing the distribution of both the F1 and AUC for each method, including the upper and lower quartiles along with the median, offer a comprehensive view of the variability and central tendency of the performance metrics across different tasks.

The superiority of CNN-MLP in both F1 and AUC across almost all tasks is not just a numerical triumph but a significant indicator of its robustness and reliability in performance. This consistent outperformance over baseline methods is attributed to the CNN-MLP’s adeptness at extracting richer semantic features from source code. By leveraging a hybrid model that combines CNN with MLP, CNN-MLP manages to capture not only the traditional features but also the semantic nuances inherent in the projects’ source codes. This ability to understand and process both traditional and semantic features gives CNN-MLP a distinct edge, enabling it to discern patterns and make classifications with a higher degree of accuracy and confidence.

The essence of CNN-MLP’s success lies in its hybrid architecture, which embodies the convergence of two powerful paradigms within machine learning. The CNN components excel at handling data with grid-like topology, such as images or spatial structures, making them adept at identifying patterns in the arrangement of code elements. On the other hand, MLPs are proficient at capturing and processing the abstract features extracted by CNNs, leading to a more nuanced understanding and processing of the source code. This synergy between CNNs and MLPs allows for a more comprehensive feature extraction process, which is critical for tasks that require a deep understanding of the data, as is often the case in software engineering and source code analysis.

The findings encapsulated in Figs. 6 and 7 underscore the effectiveness of deep hybrid models like CNN-MLP in handling complex classification tasks. This not only validates the model’s superior performance in terms of F1 and AUC but also highlights the potential of combining different neural network architectures to enhance model capability. Given these results, it is evident that hybrid models hold considerable promise for advancing the field of machine learning, offering new avenues for exploring and leveraging the unique strengths of different neural network architectures. Thus, the success of CNN-MLP in outperforming the baseline methods across a spectrum of tasks reinforces the merit of integrating diverse neural network methodologies to achieve superior performance in classification and feature extraction tasks.

To assess the statistical significance of the results and the performance difference between the proposed CNN-MLP model and the baseline model, the Wilcoxon Signed Rank Test (WSRT) and Cliff’s delta are employed. The WSRT is a non-parametric test used to determine if two paired samples originate from the same distribution, without relying on distributional assumptions. A p-value less than 0.05 indicates a statistically significant difference between the matched samples. To account for multiple comparisons, the Win/Tie/Loss indicator evaluates the performance of different models. Additionally, Cliff’s delta, a non-parametric effect size measure, quantifies the practical degree of difference between two observational data sets, complementing the WSRT analysis. The practical significance levels associated with different values of the absolute Cliff’s delta ($|{\delta }|$) are presented in Table 7.

**Table 7:** Mapping between the absolute values of Cliff’s delta ($|\delta |$) and the corresponding levels of effectiveness.

Cliff’s delta	Effective levels	Cliff’s delta	Effective levels
$0.474 \le |\delta |$	Large (L)	$0.147 \le |\delta | < 0.33$	Small (S)
$0.33 \le |\delta | < 0.474$	Medium (M)	$|\delta | < 0.147$	Negligible (N)

**Table 8:** WIN/TIE/LOSS indicators on F1 scores of TR, CNN, DBN, LSTM, DP-HNN, SDP-BB, ACGDP, and CNN-MLP.

Projects	CNN-MLP vs. TR	CNN-MLP vs. CNN	CNN-MLP vs. DBN	CNN-MLP vs. LSTM	CNN-MLP vs. DP-HNN	CNN-MLP vs. SDP-BB	CNN-MLP vs. ACGDP
jEdit-4.0	< 0.05 (+L)	< 0.05 (+L)	< 0.05 (+L)	< 0.05 (+L)	< 0.05 (+L)	< 0.05 (+L)	< 0.05 (+L)
jEdit-4.1	< 0.05 (+L)	0.136 (+N)	< 0.05 (+M)	< 0.05 (+M)	0.962 (+S)	< 0.05 (+S)	< 0.05 (−L)
Ant-1.7	0.753 (+S)	< 0.05 (+L)	< 0.05 (+L)	< 0.05 (+L)	< 0.05 (−L)	0.752 (−N)	< 0.05 (+M)
Synapse-1.1	< 0.05 (+L)	< 0.05 (+L)	< 0.05 (+L)	< 0.05 (+L)	< 0.05 (+L)	< 0.05 (+L)	< 0.05 (+L)
Synapse-1.2	< 0.05 (+L)	< 0.05 (+L)	< 0.05 (+L)	< 0.05 (+L)	< 0.05 (+L)	< 0.05 (+L)	< 0.05 (+L)
Xalan-2.4	< 0.05 (+L)	< 0.05 (+L)	< 0.05 (+L)	< 0.05 (+S)	< 0.05 (+M)	< 0.05 (−L)	< 0.05 (+L)
Xalan-2.6	< 0.05 (+L)	< 0.05 (+L)	< 0.05 (+L)	< 0.05 (+L)	< 0.05 (+L)	< 0.05 (+L)	< 0.05 (+L)
Poi-1.5	< 0.05 (+L)	< 0.05 (+L)	< 0.05 (+L)	< 0.05 (+L)	< 0.05 (+L)	< 0.05 (+L)	< 0.05 (+L)
Poi-3.0	< 0.05 (+L)	< 0.05 (−L)	< 0.05 (+M)	< 0.05 (+M)	0.883 (−N)	0.698 (−S)	0.924 (−N)
log4j-1.1	< 0.05 (+L)	< 0.05 (+L)	< 0.05 (+L)	< 0.05 (+L)	< 0.05 (+L)	< 0.05 (+L)	< 0.05 (+L)
Lucene-2.0	< 0.05 (+L)	< 0.05 (+L)	< 0.05 (+L)	< 0.05 (+L)	< 0.05 (+L)	< 0.05 (+L)	< 0.05 (+L)
Lucene-2.2	< 0.05 (+L)	< 0.05 (+L)	< 0.05 (−L)	< 0.05 (+S)	< 0.05 (+M)	0.816 (−N)	0.112 (+S)
Win/Tie/Loss	11/1/0	10/1/1	11/0/1	12/0/0	9/2/1	8/3/1	9/2/1

**Table 9:** WIN/TIE/LOSS indicators on AUC values of TR, CNN, DBN, LSTM, DP-HNN, SDP-BB, ACGDP, and CNN-MLP.

Projects	CNN-MLP vs. TR	CNN-MLP vs. CNN	CNN-MLP vs. DBN	CNN-MLP vs. LSTM	CNN-MLP vs. DP-HNN	CNN-MLP vs. SDP-BB	CNN-MLP vs. ACGDP
Xalan-2.4	< 0.05 (+L)	< 0.05 (+L)	< 0.05 (+L)	< 0.05 (+L)	< 0.05 (+L)	< 0.05 (+L)	< 0.05 (+L)
Xalan-2.6	< 0.05 (+L)	< 0.05 (+L)	0.833 (+N)	< 0.05 (+L)	0.852 (−N)	< 0.05 (−L)	0.776 (+N)
Poi-1.5	< 0.05 (+L)	< 0.05 (+L)	< 0.05 (+L)	< 0.05 (+L)	< 0.05 (+L)	< 0.05 (+L)	< 0.05 (+L)
Poi-3.0	< 0.05 (+L)	< 0.05 (+L)	< 0.05 (+L)	< 0.05 (+L)	< 0.05 (+L)	< 0.05 (+L)	< 0.05 (+L)
Ant-1.7	< 0.05 (+L)	< 0.05 (+L)	< 0.05 (+L)	< 0.05 (+L)	< 0.05 (+L)	< 0.05 (+L)	< 0.05 (+L)
log4j-1.1	< 0.05 (+L)	< 0.05 (+L)	< 0.05 (+L)	< 0.05 (+L)	< 0.05 (+L)	< 0.05 (+L)	< 0.05 (+L)
jEdit-4.0	< 0.05 (+L)	< 0.05 (+L)	< 0.05 (+L)	< 0.05 (+L)	< 0.05 (+L)	< 0.05 (+L)	< 0.05 (+L)
jEdit-4.1	< 0.05 (+M)	0.213 (+M)	< 0.05 (+M)	0.518 (−N)	< 0.05 (+M)	0.354 (+N)	< 0.05 (−L)
Lucene-2.0	< 0.05 (+M)	< 0.05 (+M)	< 0.05 (+M)	< 0.05 (+M)	< 0.05 (−L)	0.093 (−S)	< 0.05 (+M)
Lucene-2.2	< 0.05 (+L)	< 0.05 (+L)	< 0.05 (+L)	< 0.05 (+L)	< 0.05 (+L)	< 0.05 (+L)	< 0.05 (+L)
Synapse-1.1	< 0.05 (+S)	< 0.05 (+M)	< 0.05 (+M)	0.173 (+M)	< 0.05 (+S)	< 0.05 (+S)	< 0.05 (+S)
Synapse-1.2	< 0.05 (+L)	< 0.05 (+L)	< 0.05 (+L)	< 0.05 (+L)	< 0.05 (+L)	< 0.05 (+L)	< 0.05 (+L)
Win/Tie/Loss	12/0/0	11/1/0	11/1/0	10/2/0	10/1/1	9/2/1	10/1/1

Tables  8 and  9 present the results of the Win/Tie/Loss indicators for F1 scores and AUC values, respectively. These indicators are used to assess the relative performance of the proposed CNN-MLP model against other baseline models. Each column in these tables represents the p-values obtained from the WSRT and the corresponding Cliff’s delta values. The WSRT is a non-parametric statistical method to assess whether two paired samples are drawn from the same underlying distribution. If the WSRT yields a p-value less than 0.05, it signifies a statistically significant difference between the performance of the compared models. For the p-values, if the value is not less than 0.05, the original value is shown in the table. However, if the p-value is less than 0.05, it is replaced with the notation $<0.05$, indicating a statistically significant difference between the models. The tables also present the Cliff’s delta values, a non-parametric effect size measure that quantifies the practical extent of the difference between two observational data sets. As a complementary analysis to the WSRT, Cliff’s delta provides insights into the magnitude of the performance difference observed between the compared models. The practical significance level associated with the reported Cliff’s delta value is determined using Table 7. A letter denotes this level, where N represents Negligible, S for Small, M for Medium, and L for Large. Furthermore, a “+” or “-” sign accompanies the letter, indicating whether the Cliff’s delta value is positive or negative, thus signifying the direction of the observed performance difference. For example, when comparing the CNN-MLP model with the TR model on the jEdit-4.0 dataset in As shown in Table 8, the p-value is below 0.05, and the Cliff’s delta value exceeds 0.474. Referring to Table 7, a Cliff’s delta value greater than 0.474 corresponds to a Large practical significance level. Consequently, the entry “p($\delta$)” of “CNN-MLP versus TR” is represented as $<0.05$ ($+$L), indicating a statistically significant difference with a large practical significance, where the CNN-MLP model outperforms the TR model. Following the Win/Tie/Loss indicator guidelines, if the CNN-MLP model outperforms the baseline model with statistical significance, it is categorized as a “Win.” By examining the WSRT and Cliff’s delta columns, as well as the ’Win/Tie/Loss’ row in Tables 8 and 9, it becomes evident that the proposed CNN-MLP model significantly outperforms other models in most tasks, both in terms of statistical significance (p-values) and practical significance (Cliff’s delta values).

*For RQ2#*, the effort-aware evaluation presented brings an innovative angle to the analysis of defect prediction models by incorporating the dimension of effort, specifically measured through the lens of defect probability density in relation to Lines of Code (LOC). This methodology, detailed in Section “Performance measures”, places a premium on not just identifying defects but doing so in a manner that optimizes the allocation of inspection efforts. By calculating the Probability of finding Bugs in 20% of the code (PofB20) for each experiment, the evaluation provides a nuanced understanding of each model’s efficiency in prioritizing code areas that are likely to be defective.

**Figure 8:**
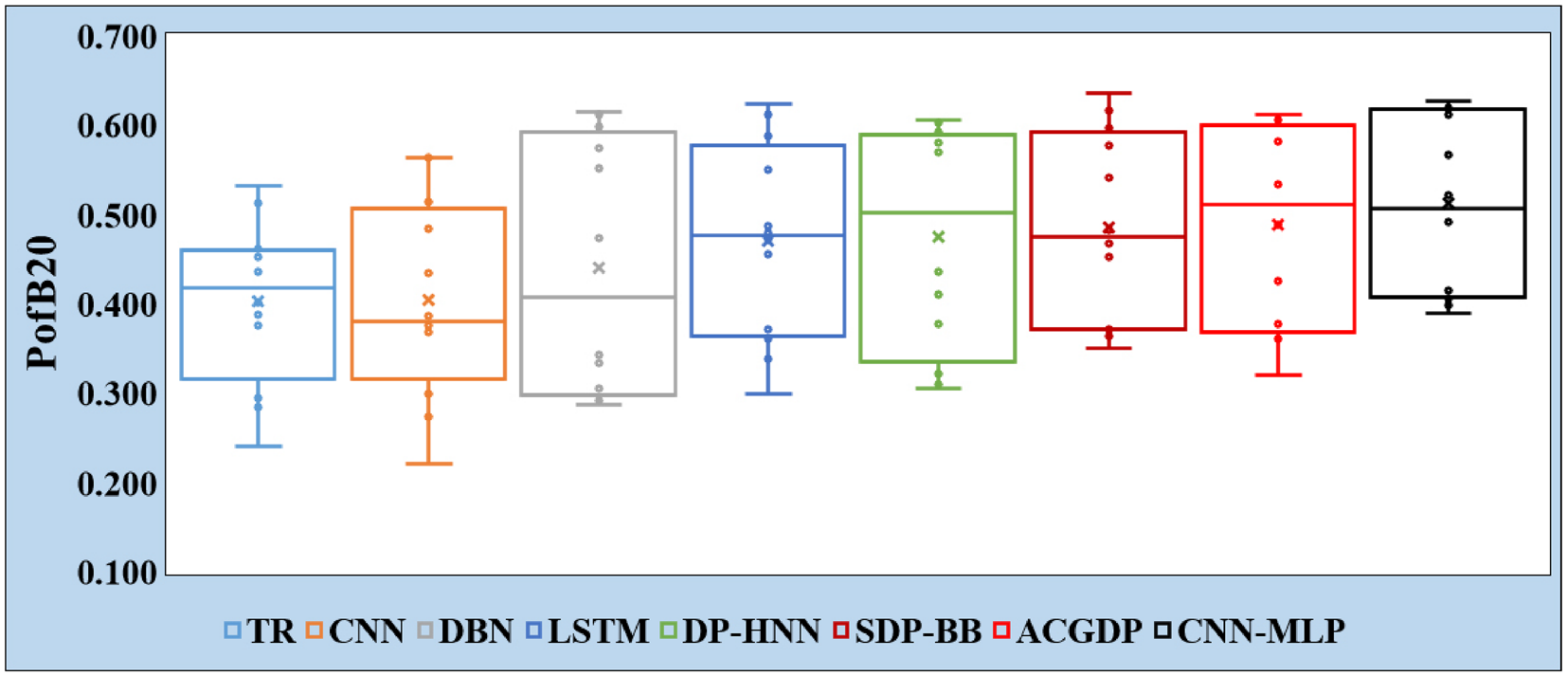
Comparison of PofB20 for TR, CNN, DBN, LSTM, DP-HNN, SDP-BB, ACGDP, and CNN-MLP.

Table 10 serves as a crucial piece of evidence in this analysis, listing the PofB20 values across 12 experiments for the CNN-MLP model and seven baselines (TR, CNN, DBN, LSTM, DP-HNN, SDP-BB, and ACGDP). The CNN-MLP model outperforms other models with an average PofB20 score of 0.513, which is superior to the next best average score of 0.489 by the ACGDP model. This achievement is highlighted in projects like Lucene-2.0 and Synapse-1.2, where the CNN-MLP scores are 0.623 and 0.521 respectively, indicating a robust capability to predict bugs efficiently compared to other models. For example, in Lucene-2.0, the CNN-MLP model not only surpasses the traditional CNN model by a significant margin (0.623 vs. 0.462) but also outperforms the more complex DP-HNN and SDP-BB models. This suggests that the integration of CNN with MLP in the proposed model helps in better feature integration and understanding, leading to more accurate predictions. Further illustrating the comparative efficiency of the models, Fig. 8 visualizes the distribution of PofB20 values across the eight models. This visualization conveys not just the average efficiency of the CNN-MLP model but also its consistency across different tasks, reinforcing the model’s reliability and effectiveness in effort-aware conditions. The emphasis on effort-aware evaluation marks a significant shift towards practical applicability in the field of defect prediction. By focusing on the PofB20 metric, this approach aligns closely with the real-world demands of software development and maintenance, where resources are limited and need to be directed intelligently. The superior performance of the CNN-MLP model in this regard suggests that its approach to extracting and prioritizing features - likely a combination of deep learning’s capability to discern complex patterns and the specific architecture’s ability to weight these effectively - is particularly well-suited to this task.

This evaluation not only demonstrates the CNN-MLP model’s effectiveness in identifying defects but also highlights its practical value in reducing the effort required to locate these defects. Such findings advocate for a broader adoption of effort-aware metrics like PofB20 in evaluating and comparing defect prediction models. They also suggest a fertile area for future research, including further refinement of the CNN-MLP model for even greater efficiency and the exploration of other models that might offer similar or better efficiency in defect prediction under effort-aware conditions.

**Table 10:** PofB20 Values of TR, CNN, DBN, LSTM, DP-HNN, SDP-BB, ACGDP, and CNN-MLP.

Projects	TR	CNN	DBN	LSTM	DP-HNN	SDP-BB	ACGDP	CNN-MLP
Xalan-2.4	0.456	0.369	0.612	0.587	0.603	0.597	0.605	**0.619**
Xalan-2.6	0.512	0.435	0.552	0.611	0.580	0.616	0.582	**0.626**
Poi-1.5	0.436	0.563	0.574	0.550	0.569	0.577	**0.584**	0.566
Poi-3.0	0.532	0.563	0.473	0.487	**0.592**	0.482	0.533	0.492
Ant-1.7	0.241	0.221	0.296	0.361	0.305	0.351	0.366	**0.406**
log4j-1.1	0.376	0.386	0.305	0.372	0.322	0.374	0.320	**0.415**
jEdit-4.0	**0.401**	0.376	0.343	0.300	0.310	0.364	0.361	0.398
jEdit-4.1	0.388	0.371	0.334	0.339	0.377	0.371	0.377	**0.389**
Lucene-2.0	0.462	0.484	0.598	0.472	0.582	0.541	0.609	**0.623**
Lucene-2.2	0.453	0.514	0.615	0.624	0.605	**0.636**	0.611	0.612
Synapse-1.1	0.295	0.299	0.287	0.455	0.411	0.452	0.426	**0.493**
Synapse-1.2	0.285	0.274	0.292	0.480	0.436	0.467	0.488	**0.521**
Average	0.403	0.405	0.440	0.470	0.474	0.486	0.489	**0.513**

To corroborate the statistical significance of the results obtained in this study, the WSRT and Cliff’s delta are employed to evaluate whether the performance difference between the proposed CNN-MLP model and the baseline models exhibits statistical significance. Table 11 presents the results of the Win/Tie/Loss indicators for the PofB20 metric. The reported indicators, including the p-values from the Wilcoxon Signed Rank Test and the Cliff’s delta values, along with their associated practical significance levels, are employed to evaluate the comparative performance of the proposed CNN-MLP model relative to other baseline models. Adhering to the guidelines for interpreting the Win/Tie/Loss indicators, when the CNN-MLP model exhibits statistically significant superior performance compared to a baseline model, it is classified as a “Win.” By examining the WSRT and Cliff’s delta columns, as well as the ’Win/Tie/Loss’ row in Table 11, it is apparent that the proposed CNN-MLP model demonstrates a significant advantage over other models in the majority of the tasks. This superior performance is observed in terms of statistical significance, as indicated by the p-values, and practical importance, as quantified by Cliff’s delta values.

**Table 11:** WIN/TIE/LOSS indicators on PofB20 values of TR, CNN, DBN, LSTM, DP-HNN, SDP-BB, ACGDP, and CNN-MLP.

Projects	CNN-MLP vs. TR	CNN-MLP vs. CNN	CNN-MLP vs. DBN	CNN-MLP vs. LSTM	CNN-MLP vs. DP-HNN	CNN-MLP vs. SDP-BB	CNN-MLP vs. ACGDP
Xalan-2.4	< 0.05 (+L)	< 0.05 (+L)	< 0.05 (+L)	< 0.05 (+L)	< 0.05 (+L)	< 0.05 (+L)	< 0.05 (+L)
Xalan-2.6	< 0.05 (+L)	< 0.05 (+L)	< 0.05 (+L)	< 0.05 (+L)	< 0.05 (+L)	< 0.05 (+L)	< 0.05 (+L)
Poi-1.5	< 0.05 (+S)	0.731 (−S)	< 0.05 (+M)	< 0.05 (+M)	0.843 (−N)	< 0.05 (+M)	< 0.05 (−L)
Poi-3.0	< 0.05 (+M)	< 0.05 (+M)	< 0.05 (+M)	< 0.05 (+S)	< 0.05 (−L)	< 0.05 (+M)	0.088 (−S)
Ant-1.7	< 0.05 (+L)	< 0.05 (+L)	< 0.05 (+L)	< 0.05 (+L)	< 0.05 (+L)	< 0.05 (+L)	< 0.05 (+L)
log4j-1.1	< 0.05 (+L)	< 0.05 (+L)	< 0.05 (+L)	< 0.05 (+L)	< 0.05 (+L)	< 0.05 (+L)	< 0.05 (+L)
jEdit-4.0	< 0.05 (−L)	0.211 (+N)	0.832 (−N)	< 0.05 (+M)	0.094 (−S)	0.744 (+N)	0.421 (+S)
jEdit-4.1	< 0.05 (+L)	< 0.05 (+L)	< 0.05 (+L)	< 0.05 (+L)	< 0.05 (+L)	< 0.05 (+L)	< 0.05 (+L)
Lucene-2.0	< 0.05 (+L)	< 0.05 (+L)	< 0.05 (+L)	< 0.05 (+L)	< 0.05 (+L)	< 0.05 (+L)	< 0.05 (+L)
Lucene-2.2	< 0.05 (+M)	< 0.05 (+M)	< 0.05 (+M)	< 0.05 (+L)	< 0.05 (+S)	< 0.05 (−L)	0.782 (−N)
Synapse-1.1	< 0.05 (+L)	< 0.05 (+L)	< 0.05 (+L)	< 0.05 (+L)	< 0.05 (+L)	< 0.05 (+L)	< 0.05 (+L)
Synapse-1.2	< 0.05 (+L)	< 0.05 (+L)	< 0.05 (+L)	< 0.05 (+L)	< 0.05 (+L)	< 0.05 (+L)	< 0.05 (+L)
Win/Tie/Loss	11/0/1	10/2/0	11/1/0	12/0/0	9/2/1	10/1/1	8/3/1

*For RQ3#* In our study, we meticulously record the time costs associated with both feature generation and training processes to provide a comprehensive understanding of the computational overhead involved. Our approach involves leveraging semantic features extracted from projects’ abstract syntax trees (AST) using Word2Vec, alongside traditional features sourced directly from the PROMISE repository.

For the generation of semantic features, the time cost encompasses two main components: the time taken to create AST nodes from the source code and the time required for the Word2Vec embedding process. Across our experiments, the time taken for generating AST nodes ranges from 0.92 to 3.5 seconds, while the Word2Vec embedding process consumes between 0.65 to 2.3 seconds. These timings provide insights into the computational resources needed for extracting semantic information from the source code, reflecting the complexity of the feature generation step.

Conversely, for traditional features sourced from the PROMISE repository, the raw data is utilized directly, bypassing the need for additional feature generation steps. This results in a more streamlined process with reduced computational overhead associated with traditional feature extraction.

Furthermore, the training time for the CNN-MLP model, incorporating both semantic and traditional features, ranges from 22 to 35 seconds. This accounts for the computational resources required for model training, including parameter optimization and gradient descent iterations.

Overall, our findings suggest that integrating semantic analysis of source code alongside traditional features and employing the CNN-MLP model as a learner holds significant promise for developing an effective and scalable Software Defect Prediction (SDP) system. The hybrid approach, combining semantic and traditional features within the CNN-MLP framework, demonstrates relevance and efficacy in improving predictive performance.

By considering both the computational costs and the performance gains achieved through the hybrid CNN-MLP approach, our study provides valuable insights into the feasibility and potential benefits of leveraging diverse feature sets and deep learning architectures for SDP tasks. These findings pave the way for further exploration and optimization of hybrid models for software defect prediction, contributing to advancements in software engineering practices.

## Conclusions and future work

This paper aims to alleviate the effort required by developers in locating defects, thereby enabling the development of high-quality software with less time and effort. We introduced CNN-MLP, a technique combining semantic and traditional features to achieve this objective. The hybrid architecture of CNN and MLP allows CNN-MLP to utilize semantic and traditional features simultaneously. A gated merging technique is employed to learn the optimal feature fusion ratio, enabling the proposed model to leverage both information types effectively. In our experiments, CNN-MLP outperformed the state-of-the-art methods regarding F1 score and AUC in non-effort-aware scenarios. Moreover, in effort-aware scenarios, CNN-MLP surpassed the baseline approaches in PofB20. The significance of CNN-MLP lies in its novel integration of CNN and MLP to address a practical challenge in software engineering code analysis and defect prediction, potentially saving software development resources while producing more reliable software.

In the future, we can extend our approach in several ways. First, we can investigate defining additional semantic features based on method-level or statement-level for Java-based language. Second, the current features can also be applied to other languages like C, C++, and Python. Additional experiments can be performed on programs from various application domains, such as Android applications. Finally, new variants of CNN and other deep models can be used to exploit their potential.

## Data Availability

We collected these projects and archived them in this link (https://github.com/ahmedabd39/promisedataset). The datasets are available from the corresponding author upon reasonable request”.
